# Heparin: The Silver Bullet of Aneurysmal Subarachnoid Hemorrhage?

**DOI:** 10.3389/fneur.2018.00097

**Published:** 2018-03-27

**Authors:** Nicolas K. Khattar, Robert F. James

**Affiliations:** Department of Neurological Surgery, University of Louisville School of Medicine, Louisville, KY, United States

**Keywords:** unfractionated heparin, neuroprotection, neuroinflammation, subarachnoid hemorrhage, delayed neurological injury, vasospasm

## Abstract

Various neurological diseases have recently been associated with neuroinflammation and worsening outcomes. Subarachnoid hemorrhage has been shown to generate a potent neuroinflammatory response. Heparin is a potential effective anti-inflammatory agent to prevent initial injury as well as delayed neurological decline. Different mechanisms of action for heparin have been proposed including, but not limited to the binding and neutralization of oxyhemoglobin, decreased transcription and signal transduction of endothelin-1, inhibition of binding to vessel wall selectins and vascular leakage into the subarachnoid space as well as direct binding and neutralization of inflammatory molecules. With a reasonably safe side-effect profile, heparin has shown significant promise in small series in human studies of aneurysmal subarachnoid hemorrhage in decreasing both initial and delayed neurological injury. Further studies are needed to validate various neuroprotective features of heparin in subarachnoid hemorrhage as well as other disease states.

## Introduction

Neuroinflammation has been recently associated with worse functional outcomes in patients afflicted by a plethora of neurological diseases, but especially in aneurysmal subarachnoid hemorrhage (aSAH) (1). Inflammation has been designated as a primary cytotoxic event affecting neurons in both acute and chronic neurological diseases. This has led to specific targeting of the inflammatory cascade (2). Unfractionated heparin (UFH) is a safe and commonly used anticoagulant in the treatment of deep venous thrombosis, pulmonary emboli, and other hypercoagulable conditions. In addition to its anticoagulant properties, UFH has been shown to have very promising significant neuroprotective anti-inflammatory properties, especially in association with subarachnoid hemorrhage (3–5). Studies have shown that heparin is prevalent in various invertebrates with no hematological system, indicating that its primary physiological role is not anticoagulation (6). Even though extrapolation to other species is not evident, the role of heparin is worth investigating. In this short review, we focus on the ever-expanding connection between heparin and neuroprotection in the setting of aSHA.

## Profile: Characteristics and Mechanism of Action

Unfractionated heparin is a highly sulfated glycosaminoglycan polymer with the most negative charge of any biological molecule (7). Inflammatory mediators are generally positively charged and binding to the heparin molecule disturbs the biochemical and electrostatic microenvironment, effectively neutralizing the actions of these mediators (8). Particularly in hemorrhagic stroke, heparin is able to bind to oxyhemoglobin and neutralize the toxic effects of free hemoglobin on other structures in the brain (9). Given the large size and uniform distribution of the negative charge along the protein, heparin can stoichiometrically bind four molecules of oxyhemoglobin, thereby quickly neutralizing the noxious effects of the oxygen free radicals on the endothelium as well as the brain parenchyma (9).

On the molecular level, heparin was found to have various neuroprotective interactions. It is able to decrease the transcription of endothelin-1 (ET-1) and the ET-1 promoter. In addition, heparin decreased the expression of the erythroid transcription factor family (GATA)-binding capacity, which is essential for ET-1 function in the endothelial cells (10, 11). ET-1 displays significant vasoconstrictive effects mediated in vascular smooth muscle cells through the epidermal growth factor receptor (EGFR) (12). Heparin-binding epidermal growth factor, a ligand of EGFR, modulates its transactivation (12). Binding of heparin to the ligand prevents EGFR receptor transactivation and was found to dampen any peri-hemorrhagic vasoconstriction and further cerebral injury in both murine and human *in vitro* models (12, 13).

Heparin and its low-molecular weight derivatives are potent inhibitors of the adhesion molecules P- and L-selectin, which mediate leukocyte rolling, the initial event governing leukocyte transmigration from vessel walls into areas of inflammation (14, 15). These mechanisms mirror effects observed in cancer metastasis, where UFH (and not other anticoagulants) was able to decrease the speed of oncologic spread by inhibiting the selectins (15). This particular effect has been further explored in cancer research and has been shown to be potent with any type of adhesion molecules (galectins, integrins, etc.), in addition to being shown to be completely unrelated to the anticoagulant properties of heparin (16). All effects were preserved with modified 2- or 6-O desulfated, N-acetylated heparins in both the oncologic and neurovascular models (16, 17). Heparin was also found to bind all the pro-inflammatory molecules such as cytokines, chemokines, as well as mediators of inflammation such as elastase and major basic protein (18–20).

Translocation of inflammatory leukocytes into an injured area through vascular leakage is a hallmark of the initiation of the inflammatory response. Presence of leukocytes in the subarachnoid space is a specific marker of inflammation (21). The accepted mechanism of action of leukocyte extravasation is through interaction of cell glycoproteins with the selectin family of proteins, generally expressed on endothelial cells (22). Heparin has been shown to specifically decrease the number of leukocytes participating in the inflammatory response to any insult to the central nervous system (23, 24).

Prior studies have also shown that microthromboembolisms may have contributed to delayed neurological deficits, therefore possibly implicating the anticoagulation effect of heparin in the neuroprotective effects observed (25). This hypothesis has been demonstrated in experimental animal models of subarachnoid hemorrhage. Mice in the experimental group showed an increase in the number of microthrombi when compared with the sham group (26). On the other hand, the advent of modifications of heparin lacking the anticoagulation domains and showing anti-inflammatory neuroprotection provides some evidence to suggest microthromboemolisms do not represent the entire pathological picture of aSHA (27). A head-to-head comparison of both forms of heparin will need to be conducted to elucidate the roles of the various domains of heparin. The various proposed mechanisms of action of UFH are summarized in Table 1.

**Table 1:** Mechanisms of action of heparin to prevent delayed neurological injury associated with subarachnoid hemorrhage.

*Direct chelation of hemoglobin in the subarachnoid space* – Heparin binds oxidized hemoglobin that is released from damaged erythrocytes. Oxyhemoglobin is believed to have a major role in the induction of vasospasm
*Decreased free-radical release* – Heparin is able to directly bind to specific molecules and inhibit the formation of free radicals through the inhibition of various pro-inflammatory molecules that contribute to their formation
*Inhibition of endothelin-1 (ET-1)* – Inhibition of mRNA transcription of ET-1 – Inhibition of transactivation of the epidermal growth factor receptor by binding of heparin to the specific ligand – Suppression of release of intracellular calcium and inositol-triphosphate in addition to ET-1 release – Inhibition of MAP-K and prevention of DNA synthesis induced by ET-1
*Prevention of K^+^ channel down-regulation induced by oxyhemoglobin release* – Downregulation of potassium channels causes a depolarization of vascular smooth muscle cells, increased incidence of calcium influxes and increased activation causing increased vasoconstriction and neurological decline
*Suppression of vascular smooth cell hyperplasia* – Smooth muscle and myofibroblast proliferation, associated with cell necrosis, lead to increased vasoconstriction, ischemia, and further neurological decline. Pathological proliferation of smooth muscle cells and neovascularization may prevent further progression of neurological injury
*Inhibition of neuroinflammatory pathways* – Inhibition of the NF-kB pathway – Binding of chemokines, cytokines, and other inflammatory proteins

## Neuroprotective Effects of Heparin in Subarachnoid Hemorrhage

Some authors hypothesize that a combination of the hemorrhage volume, vasoparalysis, and decreased cerebrospinal fluid resorption associated with aSHA triggers an increase in intracranial pressure leading to significant transient ischemia. This has been considered by various authors to be the cause of the initial neurological injury following the ictus, especially when associated with loss of consciousness at presentation (28, 29). Heparin and its non-anticoagulant derivatives have been shown to effectively counter the initial injury in various rat models of ischemia/reperfusion mimicking the initial insult following aneurysmal rupture (27, 30). The caspase pathway is triggered in the initial phase of the injury of subarachnoid hemorrhage, especially given that activation of caspase 1 was first observed in ischemic stroke (31). Countering the early activation of caspases 1, 3, 8, 9, and 11 in global cerebral ischemia can prevent neurological devastation. Animal studies demonstrated a significant decrease in cleaved caspase 3 in samples obtained from subarachnoid hemorrhage animals that were given heparin pharmacotherapy. The downregulation of apoptotic effectors leads to decreased neuroinflammation, demyelination, and decreased burden of injury (32).

The evidence supporting heparin’s anti-inflammatory role is further supported with evidence from traumatic brain injury (TBI) and chronic neurodegeneration research. Nagata et al. has shown that early administration of heparin is associated with a significant decrease in post-TBI inflammation and preservation of cognitive outcomes. These results were further supported by prior studies investigating the role of low-molecular weight fractionated heparin, enoxaparin (2, 23, 33–35). Human studies investigated the role of enoxaparin (low-molecular weight fractionated heparin) in aneurysmal SAH with mixed results (36, 37). There was a statistically significant reduction in delayed ischemia and vasospasm in the enoxaparin group in one trial but no obvious benefit in others (36, 37). A retrospective cohort study showed significant benefits of UFH in Fisher grade 3 subarachnoid hemorrhage patients (4). The patients in the heparin group had significantly less clinical and radiographic vasospasm, as well as a decrease in vasospasm-related infarction. In addition, there were a significantly higher proportion of patients who were discharged home from the hospital, instead of having to be discharged to a rehabilitation facility (4). Human studies highlighting the neuroprotective effects of heparin in various disease states are summarized in Table 2.

**Table 2:** Human studies showing neuroprotection of heparin in various neurological injuries.

*Prevention of delayed neurological injury following aneurysmal subarachnoid hemorrhage* – Low-dose IV heparin has been associated with a decrease in the rate of cerebral vasospasm (4) – Low-dose IV heparin may be associated with improved cognitive outcomes and a decrease in delayed neurological deficits (38)
*Prevention of neurological sequelae following traumatic brain injury (TBI)* – Early initiation of heparin therapy in TBI patients is associated with no neurological deterioration and decreased progression of injury on imaging (34)
*Prevention of metastasis in neoplasia* – Heparin has been associated with decrease/delay of metastasis in various cancers due to prevention of blood–brain barrier breakdown and spread of monoclonal cells into the central nervous system space (15)

In addition to the transient ischemia state associated with SAH, significant vasogenic edema and blood–brain barrier dysfunction plague this patient population and represent a dismal prognostic factor (39). Heparin has been shown to counter cerebral edema in general in various pathological states including but not limited to TBI, meningitis, ischemia, and intracerebral hemorrhages (23, 40–43). Various interactions in animal models have been reported between heparin and molecules specifically associated with cerebral edema (VEGF, bradykinin, etc.) but no proven connection linking the action of heparin on these molecules was proven.

These neuroprotective effects have been shown to correlate with a decrease in clinical vasospasm and a specific decrease in the delayed neurological injury, namely long-term cognitive decline following subarachnoid hemorrhage (32, 44, 45). Despite the fact that the number of patients included in these series remains limited; there is a positive trend and a budding interest in a closer correlation of heparin with improved cognitive outcomes. One such effort is the Aneurysmal Subarachnoid hemorrhage Trial RandOmizing Heparin, a Phase II multi-center randomized trial, studying the effects of low-dose intravenous heparin infusion on 90-day cognitive outcomes using the Montreal Cognitive Assessment test. Enrollment is expected to be complete in December 2018 (NCT02501434).

## Conclusion and Future Directions

Heparin is today a venerable, ever-young drug that has not ceased to bewilder and amaze. With its seemingly ubiquitous properties, heparin seems to show promise in various neuropathologies, with a predictable and manageable side-effect profile. Further research is needed to establish heparin as a completely safe and effective intervention in patients with subarachnoid hemorrhage. Together, with its success in other experimental neurological insults such as TBI, stroke, meningitis, cancer, etc., heparin seems to be emerging as a potential silver bullet for mitigating the delayed neurological injury commonly seen after aSHA.

## Author Contributions

RJ designed the concept of the review. NK conducted the review, compiled the information, and drafted the initial manuscript. Both authors critically reviewed and approved the final version of the manuscript.

## Conflict of Interest Statement

The authors declare that the research was conducted in the absence of any commercial or financial relationships that could be construed as a potential conflict of interest.
